# The Population Spatial Frequency Toolbox

**DOI:** 10.5334/jors.610

**Published:** 2026

**Authors:** LUIS D. RAMIREZ, FEIYI WANG, EMILY WIECEK, LOUIS N. VINKE, SAM LING

**Affiliations:** Lead Contributor, Department of Psychology, University of California San Diego, US; Department of Psychological & Brain Sciences, Boston University, US; Center for Systems Neuroscience, Boston University, US; Co-contributor, Department of Psychological & Brain Sciences, Boston University, US; Center for Systems Neuroscience, Boston University, US; Co-contributor, Department of Psychological & Brain Sciences, Boston University, US; Center for Systems Neuroscience, Boston University, US; Boston Children’s Hospital, Ophthalmology, Harvard Medical School, Ophthalmology, US; Co-contributor, Department of Psychological & Brain Sciences, Boston University, US; Center for Systems Neuroscience, Boston University, US; Department of Psychiatry, Massachusetts General Hospital, Harvard Medical School, Psychology, US; Principal Investigator, Department of Psychological & Brain Sciences, Boston University, US; Center for Systems Neuroscience, Boston University, US

**Keywords:** population spatial frequency tuning, visual cortex, fMRI, vision science

## Abstract

A goal of vision science is to develop computational models that characterize the fundamental response properties of neurons in visual cortex. One such property is spatial frequency (SF) tuning: neural populations in visual cortex selectively respond to specific bands of SF, which determines the level of detail, coarse or fine, represented from the visual input. The Population Spatial Frequency Toolbox (pSF-Toolbox) is a MATLAB package for characterizing the SF tuning of neural populations from functional magnetic resonance imaging (fMRI) data. This open-source toolbox includes stimulus presentation scripts and voxel-wise parameter optimization tools validated across a range of vision studies.

## (1) OVERVIEW

### INTRODUCTION

Neurons in the early visual cortex are selective for narrow bands of spatial frequency (SF) [1–6]. This tuning is typically characterized by two fundamental properties: a peak preference (the SF that elicits the strongest response) and a bandwidth (the range that elicits a response). These constraints effectively define the resolution limit for feature processing along the early visuocortical sheet. SF tuning is retinotopically organized as well: foveal populations prefer higher SFs and have smaller receptive fields (RFs), whereas peripheral populations are tuned to lower SFs, with larger RFs [6, 7]. This trade-off increases along the early visuocortical hierarchy; from V1–V3, peak SF preference drops while bandwidth increases [6, 8]. The functional consequence is a visual field that is centrally fine-grained but peripherally coarse.

Precise retinotopic maps of SF tuning are essential for isolating the influence of cognitive states and visuocortical conditions on SF processing (and spatial resolution) [9, 10]. However, previous human fMRI studies characterizing these maps have faced methodological trade-offs: phase-encoding (i.e., traveling wave) approaches map peak SF preference but are blind to tuning profiles, while block designs capture tuning profiles, but are too slow for the large stimulus sets required to attain voxel-wise tuning curves. To resolve this, Aghajari et al. (2020) introduced a population SF tuning (pSFT) method that circumvents these limitations, enabling efficient, voxel-wise estimation of SF tuning across and within the visual hierarchy [8].

The pSF-Toolbox presented here was developed to provide a standardized, open-source pipeline that facilitates the acquisition and estimation of pSFT. Versions of this toolbox have already been successfully deployed across multiple lines of research. For example, recent work utilized pSFT to reveal that attention sharpens bandwidths and attracts peak tuning toward the attended SF [11]. In another study, a new metric of scale invariance, “cycles per field”, was created and found to be constant across early visual cortex, confirming a fundamental assumption about human visual processing [12–15]. In tandem, preliminary research has applied pSFT to clinical questions about SF tuning under amblyopia [16], and to functional questions regarding emotional state-dependent shifts in tuning [17, 18]. These applications demonstrate this toolbox’s capacity to detect shifts in SF tuning under various conditions, and its potential in establishing a standardized, reproducible framework for characterizing visual resolution across basic, cognitive, and clinical neuroscience.

### IMPLEMENTATION AND ARCHITECTURE

The software consists of two main modules:

- measure-pSFT: Provides scripts for stimulus generation, validation, and presentation via Psychtoolbox.
- estimate-pSFT: Contains scripts for fitting the pSFT model to voxel-wise BOLD signals. There is also a sub-module, validate-pSFT, that contains scripts that demonstrate the computational validity of the pipeline.

### STIMULUS GENERATION AND EXPERIMENTAL DESIGN

**Figure 1:**
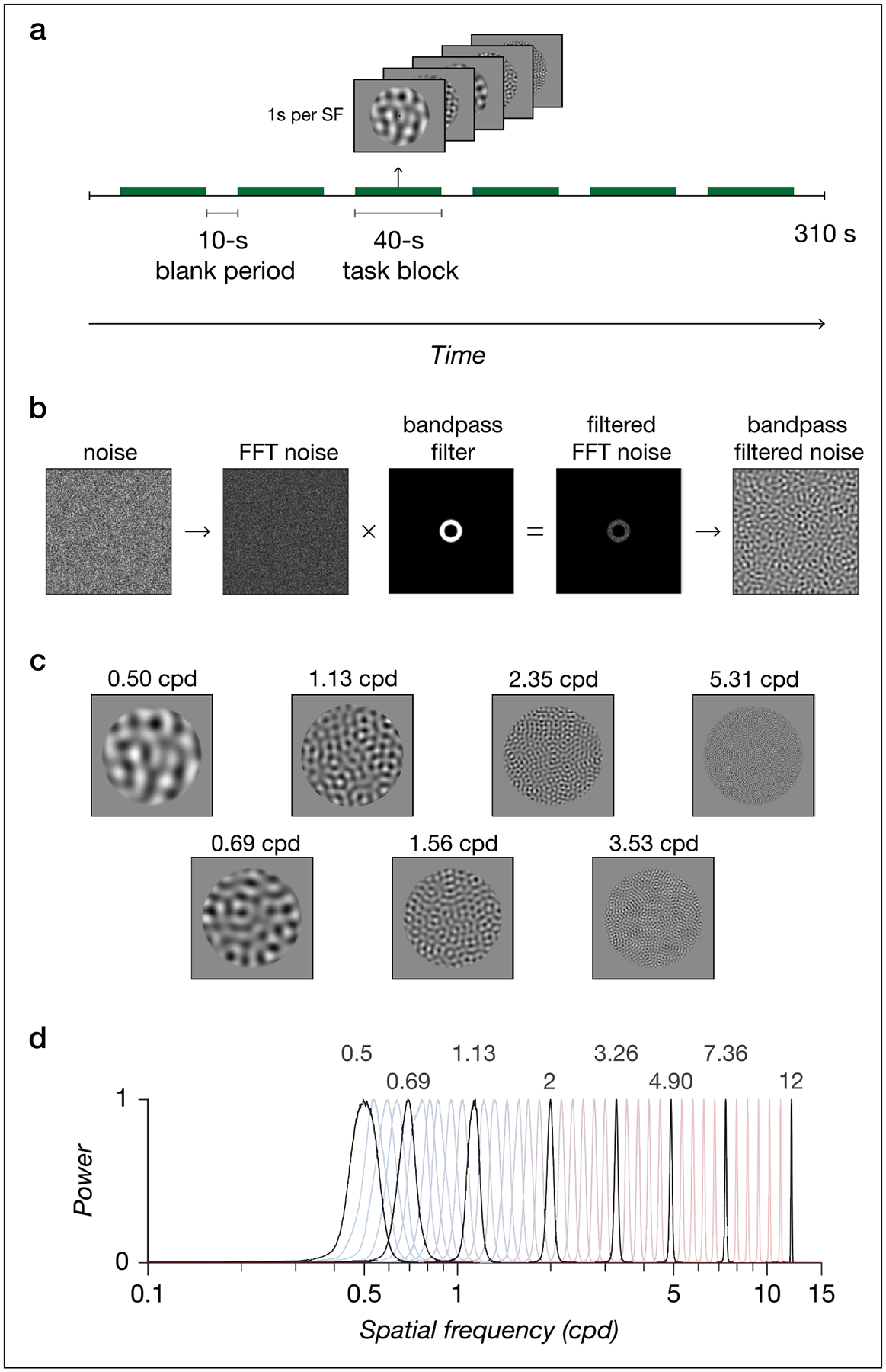
**(a) pSFT fMRI scan design.** The default scan consists of 6 40-s stimulus blocks surrounded by 10-s blank periods. In each stimulus block, 40 SF bandpass-filtered stimuli are presented in a random order while participants perform a luminance change detection task at fixation. **(b) Stimulus generation.** Every stimulus presented begins as a random sample of uniform white noise. A 2D fast Fourier transform (FFT) is performed on the noise sample, which is then masked by a circularly symmetric narrow-band filter (filter width is exaggerated here for visualization purposes). The resulting image is returned to the spatial domain and converted to 8-bit grayscale. **(c) Examples of bandpass-filtered stimuli. (d) Examples of stimulus SF energy as a function of SF.** The target SF (cpd) is provided above the peak of each energy profile.

Measuring pSFT involves presenting a sequence of SF-bandpass filtered stimuli to participants in an fMRI scanner (Figure 1a). The measure-pSFT module generates stimuli designed to drive maximal variation in SF tuning while minimizing adaptation and orientation biases. To achieve unbiased orientation content, every stimulus presented begins as a sample of uniform white noise (Figure 1b, left). In the frequency domain, white noise contains broad, isotropic energy across all orientations and SFs, the latter not ideal for driving responses of SF-selective neurons in early visual cortex (Figure 1b, ‘FFT noise’). Therefore, to isolate specific SF bands, a narrow SF-bandpass filter is applied to the 2D Fast Fourier Transform of the noise sample (Figure 1b, middle), set to a constant linear width of 0.1 cycles per degree of visual angle (cpd). To sample the SF tuning space, 40 SF-bandpass filters are created, with center frequencies that are logarithmically-spaced from 0.5 to 12 cpd (Figure 1c). The space is sampled logarithmically because SFs are represented logarithmically in human visual cortex [6].

We provide a verification function that confirms that energy exists solely in the intended SF band (Figure 1d; see /measure-pSFT/stimuli/verifyStimuli.m). This function also has the added benefit that it saves the textures to be used in an experimental session, so as long as the screen and stimulus parameters provided are identical to the experimental setup.

### EXPERIMENTAL PROCEDURE

We provide a scan session template script for data acquisition (see /measure-pSFT/run_session.m). This script should be modified to support the experimental setup of the user (e.g., screen parameters).

The script loops through a set number of scan runs, 9 by default — enough to fit within an hour of scanning. In each run, there are 6 stimulus blocks each surrounded by blank periods (Figure 1a) [8]. In short, within each task block, SF bandpass-filtered stimuli are presented in a randomized event-related design. This randomized temporal structure enables efficient estimation of the full Gaussian tuning profile (peak and bandwidth) for every voxel.

At central fixation, the dot will randomly increase in luminance. Participants should be instructed to maintain fixation and press a button when they detect a change in luminance at fixation. Run information is compiled into a structure, run_info, that should be saved. While already in run_info, the matrix containing the SF input time series for every block is stored as a separate .mat file for convenience, as the time series across multiple blocks and runs should be concatenated as an input vector (i.e., time × 1) into the pSFT optimization pipeline.

### VOXEL-WISE MODELING

For estimating pSFT parameters from fMRI data, estimatePSFT is the main high-level function, which then calls fitVoxels for voxel-wise parameter optimization.

We include an example workflow for estimating pSFT from sample_data.mat, a structure array that contains concatenated SF input and measured BOLD time series across 9 scan runs from two subjects — 100 voxels in V1, V2, and V3 (see /estimate-pSFT/example_pipeline.m) [11].

estimatePSFT takes as input the stimulus SF time series, the measured BOLD time series in percent signal change (psc), and hemodynamic response function (HRF) to return a structure array pSFT containing:

- estimated pSFT parameters (*μ*, *σ*, *β*, *β*_0_)
- estimated tuning curves
- estimated neural time series
- estimated BOLD time series
- R^2^ values
- SSE values
- fmincon exit flags

**Figure 2:**
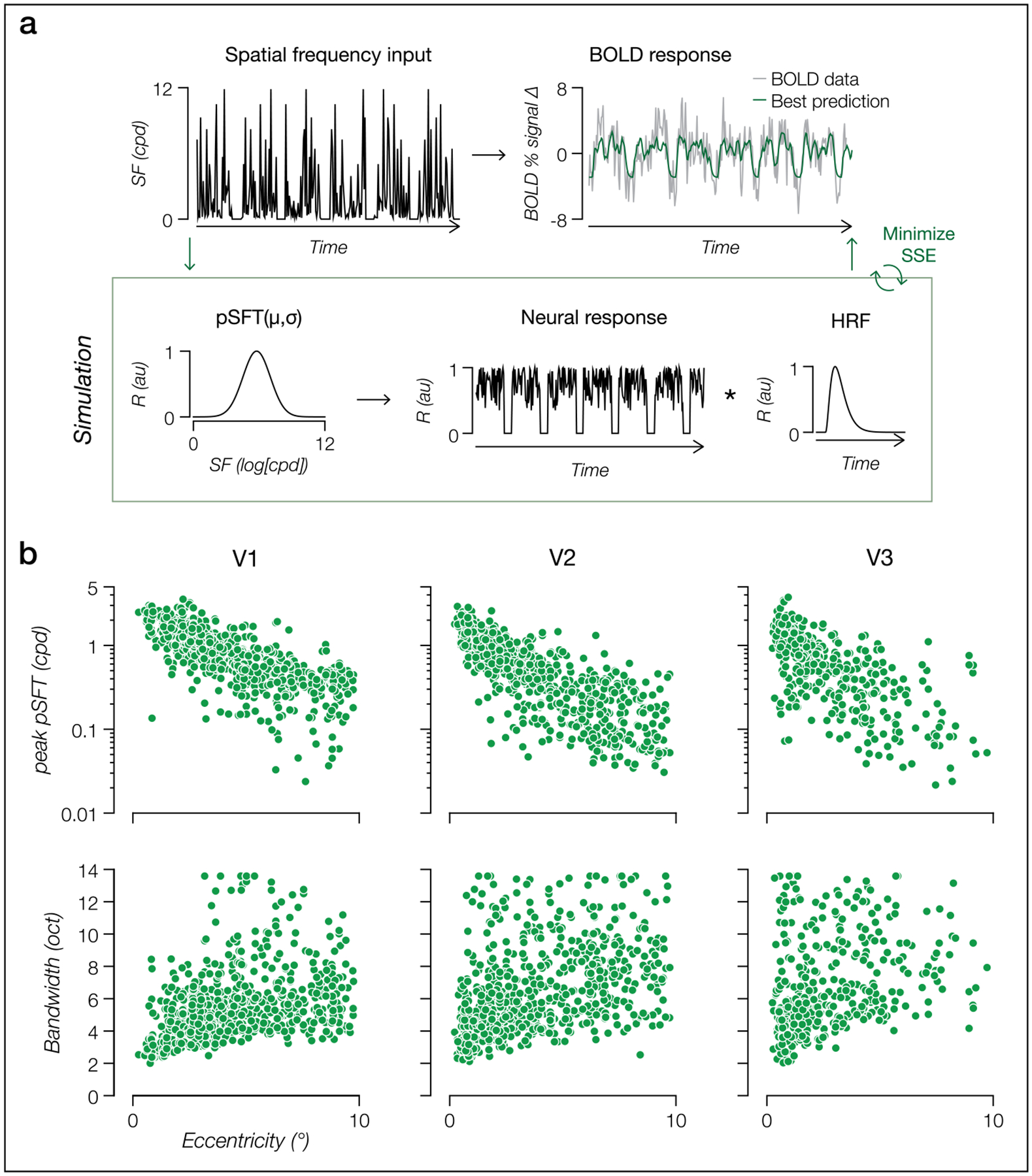
**(a) Voxel-wise estimation of population spatial frequency tuning.** A sequence of SFs is fed into a log-Gaussian function to produce a neural response. The neural response is convolved with a HRF to synthesize a BOLD signal that can be compared to the measured BOLD signal. This process is repeated and the pSFT parameters optimized until the sum of squares error (SSE) between the predicted and measured BOLD is minimized. **(b) pSFT as a function of pRF eccentricity.** pSFT and pRF parameter estimates are from a sample dataset of visual areas V1–V3 [11]. n = 200 in every subplot.

For more detail on the pSFT model itself, we model the blood oxygenation level dependent (BOLD) responses to SFs using a logarithmic Gaussian distribution [8]:

$$R([f(t)])=e^{-\frac{[\text{log}(f(t))-\text{log}[\mu ){]}^{2}}{2\sigma^{2}}}$$

where *f*(*t*) is the SF presented at time *t*, *μ* is the SF that produces the maximum response of the population (the “pSFT peak”), and *σ* is the linear SF tuning bandwidth — *μ* and *σ* being unknown. Because stimuli are not presented during blank periods between stimulus blocks (Figure 1a), the SF input during these periods is set to a small non-zero value (e.g., 0.0001) to avoid taking the logarithm of zero. The population response to a sequence of SFs, *R*[*f*(*t*)], is then convolved with a HRF, *h*(*t*), to generate a predicted BOLD signal, *B*(*t*) (Figure 2a):

$$B(t)=\beta_{0}+\beta \cdot R[f(t)]*h(t)$$

where *β*_0_ and *β* are unknown and represent the baseline and a scaling coefficient for the BOLD percent signal change, respectively. The HRF, *h*(*t*), is by default a gamma function of the form:

$$h(t)=\frac{(t/\tau {)}^{\left(n-1\right)}e^{-(t/\tau )}}{\tau (n-1)!}$$

where *τ* is the time constant (fixed to a value of 1.08), *n* is the phase delay (fixed to a value of 3), and *t* the delay between stimulus onset and the BOLD response (fixed to a value of 2.05) [19]. Voxel-wise HRFs can be provided by the user so as long as they are organized with time along the first dimension (i.e., time × voxels).

Parameters are estimated on a voxel-wise basis by minimizing the sum of squared errors using MATLAB’s fmincon optimization routine (Figure 2a).

pSFT, when analyzed in conjunction with population receptive field (pRF) estimates [20, 21], should replicate well-established relationships between eccentricity, peak preference, and tuning bandwidth (Figure 2b) [6, 8, 14, 16, 17, 11].

### pSFT VALIDATION

**Figure 3:**
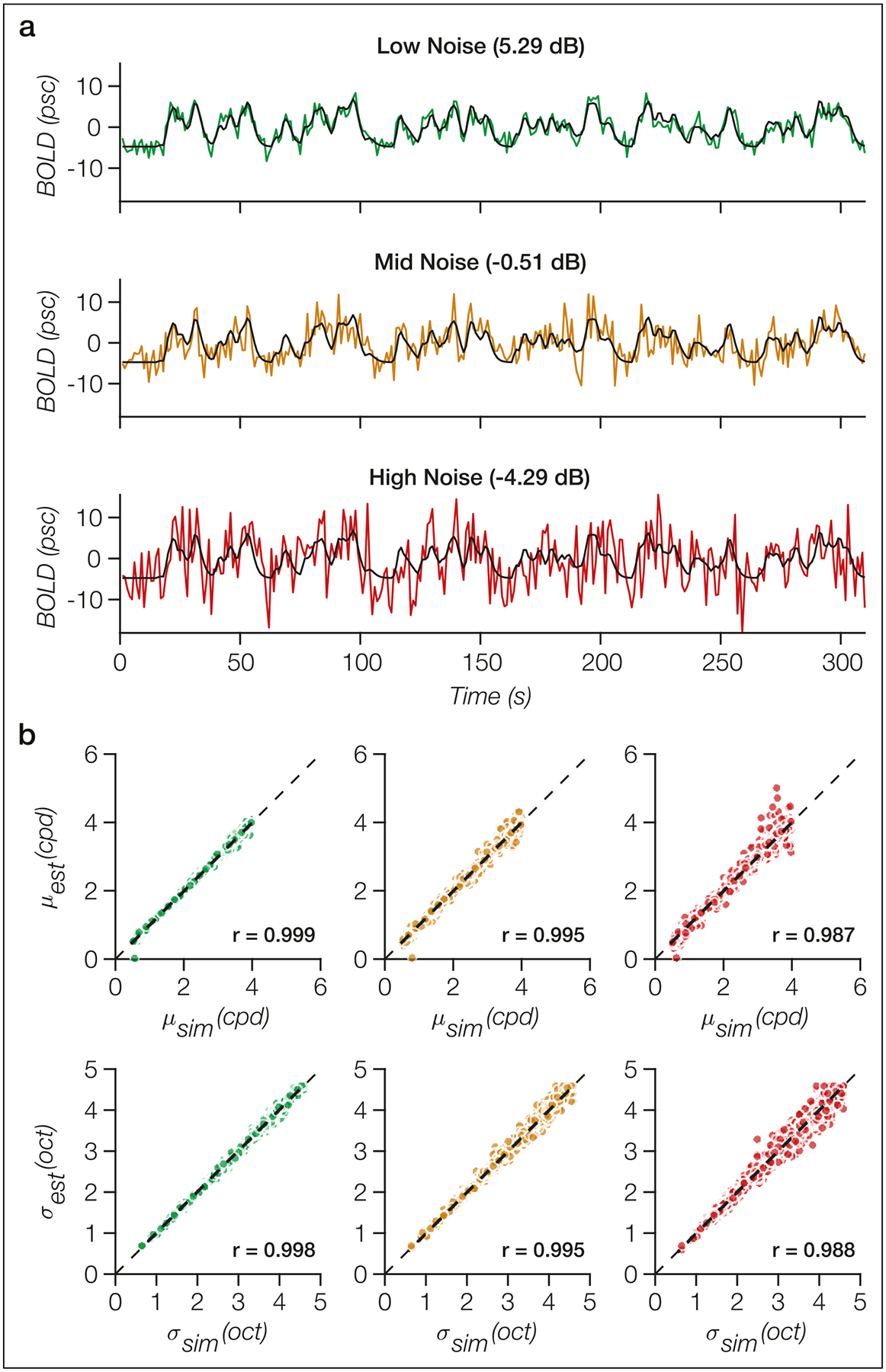
Computational Validity. **(a) Simulated BOLD.** Each subplot depicts the simulated BOLD signal of a voxel with one of three BOLD noise levels. Black curves are the clean BOLD signal, while the green, yellow, and red curves are the BOLD signal with noise. **(b) Parameter recovery**. Each subplot depicts simulated vs. estimated pSFT parameters at each BOLD noise level from 1000 simulated voxels (top row, peak; bottom row, bandwidth). The correlation coefficients are displayed on the bottom right of each subplot.

The validate-pSFT sub-module demonstrates the effectiveness of the model fitting pipeline. Inspired by the validation framework from Lerma-Usabiaga et al. (2020) [22], validate_pSFT.m generates synthetic BOLD data from known pSFT parameters across BOLD SNR levels, e.g., 5.29, −0.51, and −4.29 dB (Figure 3a). BOLD SNR_dB_ was defined as $20•{\text{log}}_{10}\left(\left.\frac{{\text{RMS}}_{\text{Signal}}}{{\text{RMS}}_{\text{noise}}}\right)\right.$. The script runs the estimation pipeline on this data and benchmarks performance by comparing recovered parameters to ground truth (Figure 3b). Metrics include RMSE and Pearson correlations with bootstrap confidence intervals. Like estimate-pSFT, this module can save timestamped results and figures.

### QUALITY CONTROL

Example data and scripts are provided to test functionality. The estimation module includes a sample pipeline that verifies required toolboxes and processes real data to validate outputs. Required and optional MATLAB toolboxes (Optimization, Parallel Computing) are detailed in the README documentation.

## (2) AVAILABILITY

### OPERATING SYSTEM

Linux Ubuntu 20.04-LTS or 22.04-LTS
MacOS 10.15.7 or later
Windows 10 or later

### PROGRAMMING LANGUAGE

MATLAB 2014b or later [23]

### ADDITIONAL SYSTEM REQUIREMENTS

- *Disk space*: Minimum 30 MB; Requirements scale with input data (and model estimates) and screen resolution (if generating stimulus textures).
- *Processor*: Multi-core recommended
- *Memory*: Requirements scale with input data (and model estimates) and screen resolution (if generating stimulus textures).

### DEPENDENCIES

- Psychtoolbox-3 [24]
- MATLAB Optimization Toolbox
- MATLAB Parallel Computing Toolbox (recommended)

### LIST OF CONTRIBUTORS

- Sara Aghajari (original framework and experimental design)
- Louis Vinke (early prototype and testing)

### SOFTWARE LOCATION

#### Archive

***Name:*** The Population Spatial Frequency Toolbox
***Persistent identifier:*** https://doi.org/10.17605/OSF.IO/BU8VE
***Licence:*** GPL-3.0
***Publisher:*** Luis D Ramirez
***Version published:*** 1.0
***Date published:*** 01/08/25

#### Code repository

***Name:*** pSF-Toolbox
***Identifier:*** https://github.com/luisdramirez/pSF-Toolbox/
***Licence:*** GPL-3.0
***Date published:*** 11/04/25

#### Emulation environment

***Name:*** N/A
***Identifier:*** N/A
***Licence:*** N/A
***Date published:*** N/A

### LANGUAGE

English

## (3) REUSE POTENTIAL

This MATLAB toolbox is intended for researchers studying visual processing with fMRI, particularly those interested in SF tuning. The code is modular and documented to facilitate adaptation for future investigations. For example, the measure-pSFT module could be adapted to investigate orientation or spatiotemporal tuning properties. Additionally, the functional pSFT maps provided by the estimate-pSFT module could serve as priors for multimodal imaging (e.g., EEG/MEG), which could help investigate the temporal dynamics of coarse-to-fine visual processing. The efficiency of the pSFT approach (~1-hour scan) makes it suitable for clinical research, providing a standardized approach for measuring pSFT in healthy or clinical populations. Users can contact the lead author via GitHub issues for questions or contributions. Support is provided through the GitHub repository’s issue tracker and documentation. Users may also contact the lead author directly at luisdramirez95@gmail.com.
